# Melanoma-Derived Extracellular Vesicles Induce CD36-Mediated Pre-Metastatic Niche

**DOI:** 10.3390/biom14070837

**Published:** 2024-07-11

**Authors:** Shankar Suman, Wendy K. Nevala, Alexey A. Leontovich, Caitlin Ward, James W. Jakub, Yohan Kim, Liyi Geng, Noah A. Stueven, Chathu L. Atherton, Raymond M. Moore, Jill M. Schimke, Fabrice Lucien-Matteoni, Sarah A. McLaughlin, Svetomir N. Markovic

**Affiliations:** 1 Department of Oncology, Mayo Clinic, Rochester, MN 55905, USA; 2 Department of Quantitative Health Sciences, Mayo Clinic, Rochester, MN 55905, USA; 3 Division of Biostatistics and Health Data Science, University of Minnesota, Minneapolis, MN 55455, USA; 4 Department of Surgery, Mayo Clinic, Jacksonville, FL 32224, USA; 5 Department of Urology, Mayo Clinic, Rochester, MN 55905, USA; 6 Department of Computational Biology, Mayo Clinic, Rochester, MN 55905, USA; 7 Department of Immunology, Mayo Clinic, Rochester, MN 55905, USA

**Keywords:** CD36, extracellular vesicles, melanoma, premetastatic niche, sentinel lymph node, macrophages

## Abstract

CD36 expression in both immune and non-immune cells is known to be directly involved in cancer metastasis. Extracellular vesicles (EVs) secreted by malignant melanocytes play a vital role in developing tumor-promoting microenvironments, but it is unclear whether this is mediated through CD36. To understand the role of CD36 in melanoma, we first analyzed the SKCM dataset for clinical prognosis, evaluated the percentage of CD36 in lymphatic fluid-derived EVs (LEVs), and tested whether melanoma-derived EVs increase CD36 expression and induce M2-macrophage-like characteristics. Furthermore, we performed a multiplex immunofluorescence (MxIF) imaging analysis to evaluate the CD36 expression and its colocalization with various other cells in the lymph node (LN) of patients and control subjects. Our findings show that cutaneous melanoma patients have a worse clinical prognosis with high CD36 levels, and a higher percentage of CD36 in total LEVs were found at baseline in melanoma patients compared to control. We also found that monocytic and endothelial cells treated with melanoma EVs expressed more CD36 than untreated cells. Furthermore, melanoma-derived EVs can regulate immunosuppressive macrophage-like characteristics by upregulating CD36. The spatial imaging data show that cells in tumor-involved sentinel LNs exhibit a higher probability of CD36 expression than cells from control LNs, but this was not statistically significant. Conclusively, our findings demonstrated that CD36 plays a vital role in controlling the immunosuppressive microenvironment in the LN, which can promote the formation of a protumorigenic niche.

## 1. Introduction

Cutaneous melanoma is an aggressive malignancy that frequently metastasizes to regional, tumor-draining lymph nodes. CD36 is a scavenger receptor that acts as a signaling receptor and a fatty acid transporter known to be involved in the progression of many cancers, including melanoma [1,2,3]. CD36 is expressed in a wide range of cells, including immune and non-immune cells, which binds with diverse ligands like thrombospondin (TSP) structural repeats, long-chain fatty acids including phospholipids, oxidized low-density lipoprotein (ox-LDL), amyloid proteins, advanced glycation end products, and molecules associated with danger- or pathogen-associated molecular patterns (DAMP or PAMPS) [1]. These ligands underpin diverse functional activities after binding with the CD36 receptor on the surface of the cells [4,5]. In the tumor microenvironment (TME), immune cells upregulate CD36 to promote lipid uptake and suppress anti-tumor immunity [6,7]. The sentinel lymph node (SLN) is defined as the first tumor-draining lymph node to receive metastasizing malignant melanocytes and exhibits features of a premetastatic niche created by melanoma-derived factors secreted into the afferent lymphatic fluid prior to metastasis [8]. Among these tumor-derived factors, high contents of oxidized lipid molecules and other immunosuppressive factors in the LN incur lymphatic remodeling [9,10]. A study shows that fatty-acid-mediated metabolic communication occurred through CD36 metabolic symbiosis between cancer cells and macrophages [11]. CD36-mediated lipid uptake enhances fatty acid oxidation (FAO) in tumor-associated macrophages (TAM) [6] as well as in myeloid-derived suppressor cells (MDSCs) to promote a protumor microenvironment [12]. FAO is also considered a driver for LN metastasis [13]. Studies show that CD36 inhibition diminishes cancer cell proliferation via modulating CD8+ T-cell and Treg-cell activities to increase anti-tumor immunity [14,15]. The immunosuppressive effects of CD36 overexpression in immune cells and other structural cells have been noted [7]. CD36 regulates mouse and human macrophage migration in response to ox-LDL [16] and plays a crucial role in fatty acid metabolism in macrophages in the TME. The binding of ox-LDL with CD36 in human macrophages plays a functional role in lipid accumulation for the development of other metabolic diseases as well [17,18]. 

Extracellular vesicles (EVs) produced from melanoma are vital in generating tumor-promoting niches [8,19]. The term “EV reception” refers more widely to the receipt of EVs [20] by macrophages, endothelial cells, stromal cells, immune cells, and cancer cells. All such cells express CD36 in tumor tissues; multiple studies have shown that CD36 contributes to the progression of many different cancers [3,21,22]. Interestingly, EV tetraspanin, CD9, can also interact with CD36 on the surface of macrophages and may have a regulatory influence on ox-LDL uptake [23]. Other immune cells, like CD8+ T cells, can mediate the generation of dysfunctional cytotoxic CD8+ T lymphocytes (CTLs) by lipid peroxidation through CD36-mediated oxidized lipid uptake [14]. These findings demonstrate the importance of CD36 targeting in TME. Previous studies show CD36 is upregulated in metastasis-associated macrophages [24] and is crucial in dysregulated immunometabolism. The lipid content secreted by melanoma directs the transformation of monocytes to tumor-promoting macrophages by altering the lipid metabolism via the upregulation of CD36 [24]. Tumor-promoting macrophages express a high level of CD36, accumulate lipids, and use FAO for energy instead of glycolysis [6]. The expression of CD36 can serve as a metabolic switch in the macrophages’ activation and differentiation, as well as mitochondrial metabolic reprogramming [1,25]. Our data from melanoma cell line (SKMEL-28 and C32TG)-derived EVs show the upregulation of CD36 when challenged with THP1. It has been demonstrated that tumor-derived EVs induce a glycolytic-dominant metabolism in immunosuppressed macrophages [26]. Nevertheless, the role of tumor-derived EVs in inducing CD36 expression in the recipient cells is not understood, which may directly impact the deregulation of lipid metabolism. Therefore, our study is highly relevant in understanding this critical question of whether melanoma EV mediates the alteration of CD36 in recipient cells to mediate the development of the premetastatic niche. 

## 2. Materials and Methods

### 2.1. Clinical Specimens

All clinical biospecimen (lymphatic fluid and LN tissues) data were acquired in accordance with the Declaration of Helsinki and approved by the Mayo Clinic’s Institutional Review Board. Given the study’s retrospective, minimum-risk design, the IRB withheld informed written consent from participants. The lymphatic fluid of melanoma patients downstream of primary cutaneous melanomas (*n* = 5) and controls (*n* = 5 from the non-malignant post-operative fluid collected from the lymph node dissection field) was collected as described earlier [19]. LN tissue biopsies from two subjects of each type (SLN (+), SLN (−), and control LN) were collected to perform MxIF staining. 

### 2.2. Cell Lines, Cell Culture, and CD36 siRNA Transfection

Human melanoma cell lines (C32TG and SK-MEL28) and human monocytic cell line (THP1) were purchased from the American Type Culture Collection (ATCC), USA. We followed the recommended procedures from ATCC for subculturing these cells. Human lymphatic endothelial cells (HLECs) were purchased from ScienCell Research Laboratory, Carlsbad, CA, USA, and the recommended endothelial cell medium was used for culturing these cells. SiRNA against CD36 was purchased from Origene, Boston, MA, USA, which included three CD36 SIRNA duplex (20 μM) (duplex sequences: SR319610A rCrArArCrCrUrArUrUrGrGrUrCrArArGrCrCrArUrCrArGAA, SR319610B rGrGrCrCrUrGrArUrArGrArArArUrGrArUrCrUrUrArCrUCA, and SR319610C rGrGrArUrUrArArArCrCrCrArArArUrGrArArGrArArGrAAC) and non-targeting scrambled (NTC) siRNA used as a negative control. We used lipofectamine (Invitrogen by Life Technologies, Carlsbad, CA, USA) for transfection with C32TG and SKMEL28 cells by following the manufacturer’s instructions. 

### 2.3. EVs Collection from Melanoma Cell Lines and Human Lymphatic Fluid

For the lymphatic EV collection, lymph channels were surgically excised, and effluents of channels dissected from LN were utilized following the procedure published earlier [19,27]. Briefly, the patient’s tumor-draining LN was identified during SLN mapping using methylene blue dye. The afferent channel proximal to the SLN was clipped on both ends before dissecting and transported to the laboratory. The surgical clip was removed, and the LN channel was perfused with 30-gauge needle with RPMI media and filtered by a 0.8 µm syringe filter. The effluent was used for LEV collection using size-exclusion chromatography with qEV1 column (70 nM) (IZON). Control lymph channels were collected from women undergoing prophylactic mastectomy with no evidence of cancer. Due to the lack of availability of large amounts of LEVs from human subjects, melanoma-cell-based EVs were used for molecular studies. Melanoma cell lines were cultured in hypoxic conditions using a Petaka G3 low-oxygen transfer flask for melanoma-derived EV collection. We further used exosome-depleted FBS (Gibco, Grand Island, NY, USA) to culture the cells. Culture media from the melanoma cells were harvested after 72 h of cell culture and centrifuged at 2100× *g* for 15 min at 4 °C to remove the debris, and we further used XPN-90 Ultra Centrifuge to collect EV pellet, with rotor: 70 Ti, speed: 100,000× *g*, and time 3 h at 10 °C. 

### 2.4. EV Labeling and Treatment with Cells

We followed a previously published method to stain melanoma EVs with carboxyfluorescein diacetate succinimidyl ester (CFSE) dye [28]. THP1 or HLEC cells were challenged for 24 or 48 h to analyze the effect of EVs. For the CFSE-labeled EV tracking analysis, 1 × 10^10^ freshly labeled EVs were incubated with 1 × 10^6^ HLEC or THP1 cells. EV-staining and treated cells were initially observed using fluorescent microscopy. Further, ImageStream analysis of CFSE-labeled EVs treated with THP1 cells was carried out. Western blot of unlabeled-EV-treated cells was performed separately (see next section). To analyze the effects of melanoma EVs in CD36 expression and macrophage polarization, we treated PMA-induced M0 macrophages of THP1 cells and added melanoma-derived EVs for 48 h. Positive control for M1 macrophage (LPS + IFN-γ) and M2 macrophage (IL4) was also used to compare the effects of the melanoma EVs. Flow analysis was carried out with CD36, M1 macrophage (HLA-DR), and M2 macrophage (CD163, CD206) markers to analyze the impact of EVs on challenged THP1 cells.

### 2.5. Western Blotting

Cell lysates were first quantified by a BCA protein quantitation kit (Thermo Fisher, Waltham, MA, USA) for western blotting. Equal amounts of protein from cell lysates were mixed with 2× loading dye in SDS-PAGE gel and run with an electrophoresis unit until the tracking dye reached the bottom. Separated proteins in SDS-PAGE gel were transferred to PVDF-activated membranes using a trans-blot transfer system (Bio-Rad, Hercules, CA, USA). Transfer of proteins in PVDF was confirmed with Ponceau S staining. Membranes were blocked with 5% non-fat milk in a 1× TBST solution. Further, primary antibodies against CD36 (18836-1-AP, Proteintech, Rosemont, IL, USA) and beta-actin (20536-1-AP, Proteintech) were added overnight at 4 °C under a constant shaker. Recombinant Human CD36 Protein was loaded for CD36 positive control (catalog: 10752-H08H, Sino Biological, Chesterbrook, PA, USA). HRP-conjugated secondary antibody was added for 2 h at room temperature after removing primary antibodies. HRP substrates were used to develop the blots. Between the steps, the membranes were washed thrice with 1× TBST buffer to remove extra unbound antibodies. Blots were developed using chemiluminescent substrates (Bio-Rad) and imaged on X-ray films, further analyzed by Image J software ver1.53. 

### 2.6. Flow Cytometry

#### 2.6.1. EV Flow Analysis

Human-lymph-node-derived EVs (LEVs) and melanoma-cell-line-derived EVs were analyzed by an A60-MicroPlus nanoscale flow cytometer (Apogee Flow Systems Inc., Hertfordshire, UK). Before sample analysis, the A60-MicroPlus was calibrated using a Rosetta calibration bead mix (Exometry Inc., Amsterdam, The Netherlands) as described [29]. The side scatter triggering threshold was set at 2300 a.u, corresponding to a scattering cross-section of 19 nm^2^ and a particle diameter of 188 nm (Refractive Index core = 1.38; Refractive Index shell = 1.48; and shell thickness = 4 nanometers). To determine the concentrations of CD36-positive EVs, samples were diluted in sterile PBS and were run at a flow rate of 1.5 μL/min for 1 min with an event rate below 7000 events per second to avoid the swarm effect. The samples were labeled with anti-CD36 antibody (Cat#336202, Bio Legend, San Diego, CA, USA) conjugated with Alexa fluor 647 labeling kit (Thermo Fisher Scientific, A20186). Before each run, nFCM underwent a quality control procedure, including a run with a mix of polystyrene and silica polydisperse beads (Apogee bead mix #1493, Apogee Flow Systems) to control for instrument sensitivity and flow rate stability. Buffer-only control (sterile PBS) and antibody-only samples in sterile PBS were analyzed with the same instrument/acquisition settings, and the event rate was kept below 80 events per second. Data analysis was performed in FlowJo version 10.6.1.

#### 2.6.2. Cellular Flow Cytometry and ImageStream Analysis

We followed previously published methods for the analysis of flow and ImageStream analysis [27]. Briefly, following incubation of melanoma EVs with THP1 cells, these cells were stained with surface markers HLA-DR, CD163, CD206, and CD36 (Bio Legend), and data were acquired with BD LSRFortessa™ Cell Analyzer. We used FlowJo to analyze the data. For flow-imaging analysis, melanoma-derived EVs were extracted and fluorescently labeled, as mentioned above. The EV-challenged THP1 cells were used for data acquisition using ImageStream MKII imaging flow cytometer (Amnis, Seattle, WA, USA) and which were analyzed with Ideas 6.2 software (Amnis).

### 2.7. Real-Time PCR

We examined the CD36 gene transcripts in EVs collected from melanoma cell lines using real-time PCR. Briefly, cell-based EV pellets were lysed, and total RNA was collected using Qiagen RNeasy plus kit, and further synthesis of cDNA from total RNA (100 ng) was performed using Reverse-Transcription Kit following manufacturer’s instructions (Applied Biosystems, Foster City, CA, USA; Catalog: 4368814). The QuantStudio^TM^ 3 Real-Time PCR system was used to perform quantitative real-time PCR in optical 96-well plates (Applied Biosystems) with 20 μL reaction volumes containing 10 μL of PCR master mix (Taqman 2× Universal PCR master mix, Applied Biosystems, Catalog: 4444963). The settings for qPCR cycling were as follows: 10 min at 95 °C, 40 cycles of 15 s at 95 °C, and 60 s at 60 °C. Applied Biosystems QuantStudio 3.0 Real-Time PCR Software automatically determined the threshold fluorescence level. We purchased probes from Thermo Fisher Scientific (GAPDH: Hs02786624_g1, CD36: Hs00354519_m1) to conduct TaqMan Gene Expression analysis. The expression levels of GAPDH were used to standardize the results. 

### 2.8. Bioinformatics and Statistical Analysis

Kaplan–Meier survival curves were estimated, and the log-rank test was performed using the TCGA melanoma (SKCM) dataset (*n* = 481), and the UCSC Xena database was used to determine the progression-free survival by grouping values in the upper and lower quartiles of CD36 expression [30]. CD36 biological functions with hallmark concept (hallmark enrichment plot) were generated with core cancer hallmark gene set (*n* = 1574) from CancerHallmarks.com. The gene correlation of CD36 with other genes of interest and the correlation of CD36 with immune infiltration in tumors from the SKCM dataset was analyzed using Timer 2.0 web server [31]. TIMER2.0 was used to interpret the estimated immune infiltration with CD36 expression through EPIC, CIBERSORT-ABS, and MCPCOUNTER methods. For analysis of %CD36 expression in EVs among patient control sample analysis, Welch’s *t*-test was used with a *p*-value < 0.05, which was considered significant. Statistical model analysis of MxIF spatial data is detailed in the next section. 

### 2.9. Spatial Multiplexed Immunofluorescence (MxIF) Staining and Analysis

We followed our published method with slight modifications [32] for spatial MxIF staining and analysis. Briefly, we utilized Cyclic MxIF to visualize multiple stains within selected tissue sections of normal LN, SLN (−), and SLN (+). This technique involves a repeated staining cycle with specific antibodies and a bleaching step after each staining to inactivate the fluorescent dyes. Slides were stained with antibodies for 1 h in a dark, humidified chamber at room temperature. Following staining, the slides were washed three times with PBS to remove unbound antibodies. After washing, the slides were coverslipped in preparation for imaging with the InCell imager by GE HealthCare (Chicago, IL, USA). After imaging, the coverslips were removed using warm PBS to initiate bleaching. The bleaching solution, consisting of 0.5 M sodium bicarbonate (pH 11.2), hydrogen peroxide, and DI water, was used to inactivate the fluorescent dyes. Slides were incubated in bleaching solution two times for 15 min at room temperature. Following the bleaching, slides were washed three times in PBS to remove any residual bleaching solution. A DAPI recharge step was conducted to restore nuclear staining, after which the slides were coverslipped again for final imaging. This comprehensive cyclic procedure ensures detailed visualization of multiple stains within a tissue section, as described previously [32]. We used core biomarkers with myeloid and lymphoid panels (DAPI, CD14, CD15, CD16, CD68, CD163, CD206, CD11b, CD11c, CD209, CD38, CD31, CD36, CD3, CD4, CD68, CD8, NaKATPase, MPO, FOXP3, PDL1, FAP, and FASN) to leverage pixel classification (Table S1). 

The INCell Analyzer 2500HS (GE Healthcare, Chicago, IL, USA) captured an entire slide image of the LN tissue using DAPI and Cy3 fluorescent channels to create virtual H & E stains. The virtual H & E images were utilized to create fields of view (FOVs) in areas of interest. The analysis included six lymph nodes in the three categories mentioned above. Fifteen to twenty-five FOVs were selected in each lymph node depending on the overall size of the tissue. Annotation and classification of the markers in 36 FOVs from six patients (5–6 FOV/patient) were carried out manually in QuPath. A spatial logistic mixed effects model [33] was used to estimate the probabilities of cells expressing CD36. The outcome in this model was cell-level binary classification of CD36 expression. Fixed effects in the model differentiate between the three categories (normal LN, SLN (−), and SLN (+)). Subject- and FOV-specific random effects are included to account for repeated FOVs per subject and FOV-level heterogeneity. Spatial correlation (clustering of CD36 cells may make nearby cells more likely to express CD36) was included with a spatial random effect using a squared exponential correlation function. For CD36 colocalization analysis, a similar spatial logistic mixed model was used. The colocalization model uses the outcome of the proportion of cells in a 40-micron radius around each CD36 of a specific cell type. Fixed and random effects are specified identically to those in the previous model. Separate models were run to assess the outcomes of colocalization of CD36 cells with other CD36 cells, as well as cells expressing CD3, CD4, CD8, CD14, CD11C, CD16, PDL1, FOXP3, CD209, FASN, CD11B, CD163, CD68, and MPO. The False Discovery Rate procedure was used to adjust *p*-values for multiple testing [34]. In particular, the model estimates high variability between FOV, leading to large confidence intervals. 

## 3. Results

### 3.1. High CD36 Expression Is Associated with Melanoma Progression

**Figure 1:**
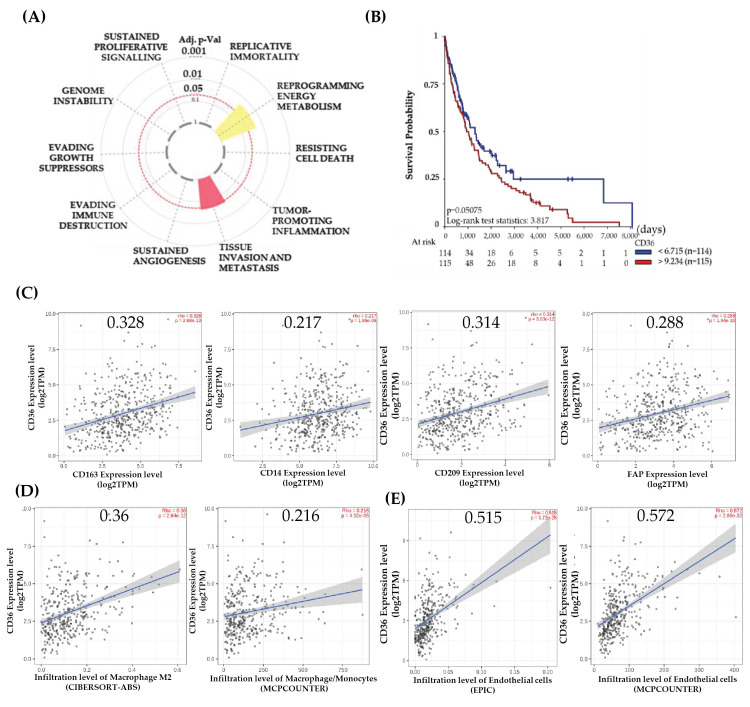
Role of CD36 in the clinical prognosis and immune infiltration correlation in cutaneous melanoma: (**A**) Hallmark enrichment plot of CD36 generated with core cancer hallmark gene set (*n* = 1574) shows that CD36 is associated with invasion and metastasis of melanoma as well as reprogramming energy metabolism (*p* < 0.05). (**B**) Progression-free survival with CD36 expression in melanoma patients, using upper (red) and lower (blue) quartile data from the SKCM dataset. (**C**) Correlation of CD36 with CD163, CD14, CD209, and FAP in SKCM dataset (*n* = 471). (**D**) CD36 positively correlates with the infiltration level of M2 macrophages in the SKCM-metastasis dataset (*n* = 368). (**E**) Correlation between CD36 and infiltration level of endothelial cells in SKCM-metastasis dataset (*n* = 368).

CD36 is a widely reported molecule for its role in cancer progression and is overexpressed in many cancers (Figure S1). From the core integrated hallmark gene dataset, the CD36 hallmark plot indicates a significant association of CD36 with the reprogramming of energy metabolism and tissue invasion and metastasis (Figure 1A). To identify the clinical significance of CD36 expression in melanoma, we analyzed the progression-free survival (PFS) in the SKCM dataset, choosing the upper and lower quartiles of CD36 expression. We found that melanoma patients who had a high CD36 expression in the tumors progressed significantly sooner than patients whose expression of CD36 falls in the lower quartile (log-rank test statistics = 3.817, *p* = 0.05) (Figure 1B). We used the TIMER-2 gene module to correlate CD36 with the cellular markers and immune infiltrate analysis in TCGA-SKCM melanoma datasets. The analysis with this dataset shows a positive correlation of CD36 with CD163, CD209, CD14, and FAP (Figure 1C). Moreover, M2 macrophage infiltration in the CD36 overexpressing tumors is more positively correlated compared to monocyte (Mono) and M0/M1 macrophages (Figure S2). Moreover, metastatic tumors express more CD36 than primary tumors (Figure S2). The dataset of SKCM-metastasis indicates the higher infiltration of M2 macrophages (CD163) and endothelial cells with a significant positive correlation (Figure 1D,E). These data may suggest that metastatic melanoma upregulates CD36 in tumors and surrounding endothelial cells and macrophages for cancer progression. Endothelial cells and macrophages are among the critical cells in the LN that serve as the recipients of lymph-associated melanoma secretory factors that mediate SLN remodeling and develop a premetastatic niche [35,36]. 

### 3.2. Melanoma-Secreted EVs Carry High Levels of CD36 Cargoes, Which Upregulate Their Expression in the Recipient Human Monocytic and Endothelial Cell Lines

The SLN microenvironment becomes immunotolerant, thereby transforming into a premetastatic niche for primary cutaneous melanoma. The detailed mechanisms of this change are still poorly understood. However, emerging evidence suggests a role for tumor-derived EVs as carriers of immune-modulating factors to suppress SLN immunity [8]. 

**Figure 2:**
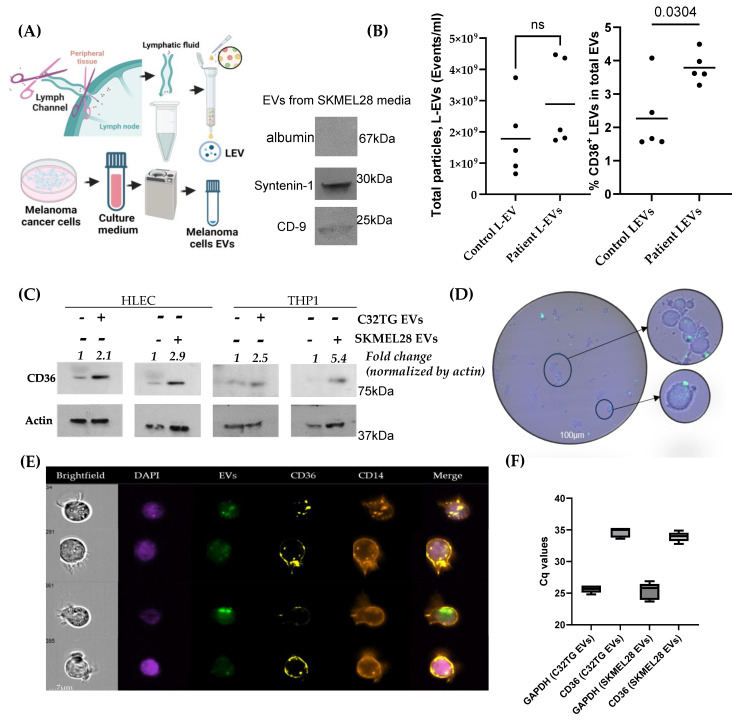
Melanoma-derived EVs upregulate CD36 expression in the recipient cells. (**A**) Brief procedure for LEV and melanoma-cell-derived EV collection and characterization. Summarily, afflicted afferent lymph channels were surgically excised, and effluents of channels were utilized to collect LEVs through size-exclusion chromatography. Cell line culture-conditioned media was used to collect EVs by ultracentrifugation. All collected EV characterizations were carried out with NTA (see method in Section 2.3 for more details). (**B**) LEVs collected from patients possess a significantly higher percentage of CD36 + EVs in total LEVs than control LEVs. (**C**) SKMEL28 and C32TG melanoma-cell-derived EVs increase expression of CD36 in HLEC (endothelial) and THP1 (monocytic) cells after 24 h of exposures. (**D**) Microscopic observation of CFSE-labeled-SKMEL28-derived EVs in HLEC cells (scale bar: 100 µm). (**E**) ImageStream analysis of CFSE-labeled-SKMEL28-derived EVs in THP1 cells (scale bar: 7 µm). (**F**) The box plot indicates the real-time PCR data by Cq values of CD36 and GAPDH gene expression in EVs collected from melanoma cell lines. Original images of (**A**,**C**) can be found in Supplementary Materials.

We first determined whether EVs of melanoma patients and the respective control from lymphatic fluid alter the expression of CD36. We used nanoscale flow cytometry and analyzed the %CD36 in total LEVs from melanoma patients and control (Figures S3 and S4). Our data indicated that EVs in the patient LEVs expressed a significantly higher %CD36 than control LEVs (Figure 2B). For in vitro mechanistic experiments, melanoma-derived EVs were collected from melanoma cell lines SKMEL28 and C32TG. To mimic the natural tumor hypoxia conditions, these EVs were cultured in the hypoxia chamber, using exosome-free FBS to avoid the impact of contaminated EVs. Furthermore, THP1 and HLEC cells were treated with melanoma-derived EVs for 24 h, and we detected the upregulation of CD36 for both human monocytic-THP1 and human LN endothelial cells with an approximately two- to five-fold change (Figure 2C). The upregulation of CD36 in THP1 cells was consistent in 48 h as well, and we found an upregulation in SKMEL28 cells (Supplementary Figure S5). To analyze the EV entry to recipient cells, we labeled them with CFSE dye before treating them with recipient cells. We used THP1 and HLEC cells to investigate the effect of EVs upon treatment with these cells with a ratio of ~10^4^ EVs per cell. A microscopic examination of HLEC cells detected the appearance of CFSC-labeled EVs either on the surface of the cells or internalized within the cells (Figure 2D). Further, we analyzed the THP1 cells challenged with CFSE-labeled SKMEL28 EVs by ImageStream imaging flow cytometer, and we acquired five single channels (Brightfield, DAPI, CD14-PECy7, CD36-PE, and EVs-FITC). We detected the internalized EVs with CD36 in the THP1 cells, demonstrating that melanoma EVs could be important factors in upregulating CD36 in monocytic cells (Figure 2E). EVs from these melanoma cells showed evidence of CD36 mRNA cargo (Figure 2F). Further, we also detected an upregulated CD36 expression in the HLEC after the incubation of SKMEL28 EVs (Figure S6). 

### 3.3. SKMEL28-Derived EVs Alter Macrophage Polarization Derived from THP1 Cells

**Figure 3:**
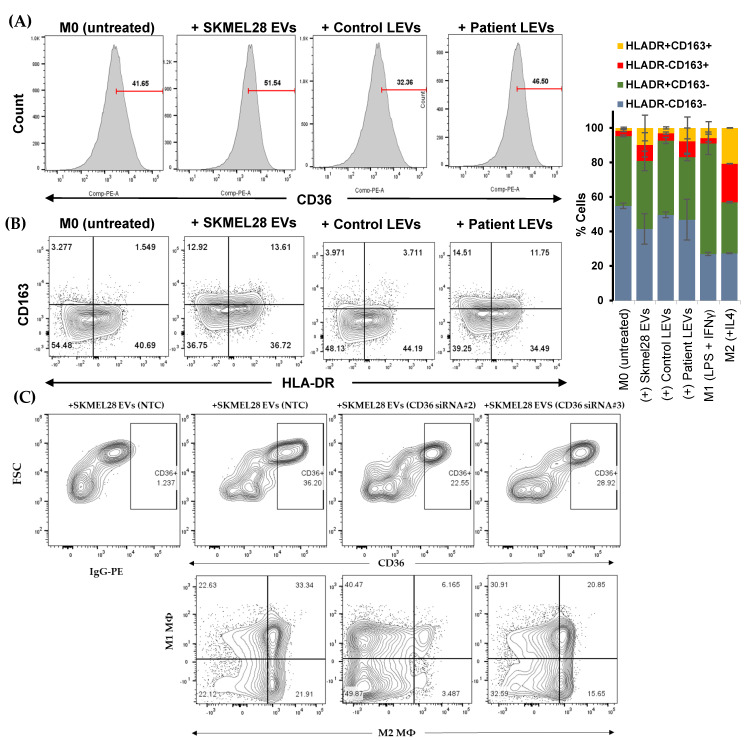
Melanoma-derived EVs regulate M2-macrophage-like characteristics by upregulating CD36. (**A**) CD36 surface expression was increased upon EV exposure to cells compared to the control in M0 macrophages derived from THP1 cells. (**B**) Melanoma-derived EV exposure increases M2-macrophage-like characteristics by upregulating CD163 in PMA-pretreated THP1 cells. (**C**) EVs collected from CD36-silenced SKMEL28 cells decrease M2 macrophage characteristics in THP1-treated cells through CD36 downregulation.

Macrophages are essential regulators of tumor immunity, especially for creating a protumorigenic microenvironment. To determine the impact of EV exposure on macrophage polarization, we used THP1 cells and transformed them into M0 macrophages by treating them with PMA. Further, we introduced melanoma-cell-line-derived EVs to M0 macrophages. M0 macrophages were exposed to M1/M2 polarizing factors for 48 h as positive controls. We found that patient-derived LEVs increased CD36-expressing populations in THP1 cells compared to control LEVs; in addition, the CD36 expression was also increased in SKMEL28-derived-EV-treated cells compared to untreated cells (Figure 3A and Figure S7). M0 macrophages treated with EVs alter their phenotypes, especially with HLADR and CD163; we found that CD163 and HLADR populations were increased after challenging with EVs, and HLADR^+^CD163^+^ cells were detected in the patient or melanoma-cell-line-EV-challenged cells, as well as IL4-treated M0 macrophages (Figure 3B). This shows that M0 macrophages treated with EVs develop into the population of cells with a high CD36 expression and fall more towards M2 macrophages with flow cytometry (Figure 3A,B).

#### CD36 Knockdown in SKMEL28 Impair Melanoma-Cell-Line-Derived-EVs Induced M2 Macrophage Polarization in THP1 Cells

We also re-analyzed the macrophage polarization of EVs collected from siRNA-directed CD36-silenced melanoma cells to observe the polarization effect with CD36 carrying EVs versus non-CD36 carrying EVs. CD36-siRNA-transfected SKMEL28 cells show an approximately two-fold decrease in CD36 protein levels compared to non-targeted control (NTC) siRNAs (Figure S8). The EVs collected from melanoma cell lines transfected with NTC and siRNA show a difference in the total mRNA levels. EVs from CD36-silenced SKMEL28 cells show up to an approximately ten-fold downregulation of mRNA cargo in the EVs compared to NTC-transfected cells by a real-time PCR analysis (Figure S9). Using flow-cytometry-based analysis, EVs derived from CD36 siRNA knockdown cell lines and NTC-siRNA-transfected cells show a reduced CD36 surface expression on the THP1-treated cells from 36% to the lowest ~22.5% (Figure 3C). To analyze whether the CD36 expression in EV cargoes can influence macrophage polarization, we also analyze M1 macrophages (HLADR) and M2 macrophage markers (CD163 and CD206). Our data show that the melanoma cell’s (SKMEL28) EV-mediated CD36 downregulation affects the THP1 cell polarization. We detected a decrease in the combined CD163 and CD206 expression in the macrophages treated with EVs from CD36-siRNA-transfected melanoma cells compared to EVs from NTC-transfected melanoma cells (Figure 3C). This result demonstrates that the extent of M2 macrophage polarization is correlated with the amount of CD36 cargoes in the EVs challenged with M0 macrophages (derived from THP1 cells). The M2 macrophage marker (CD163 and CD206) expression was also corroborated in CD36-silenced-C32TG-cell EVs (Figure S10). 

### 3.4. CD36 Is More Likely to Colocalize with Myeloid Cells in SLNs

**Figure 4:**
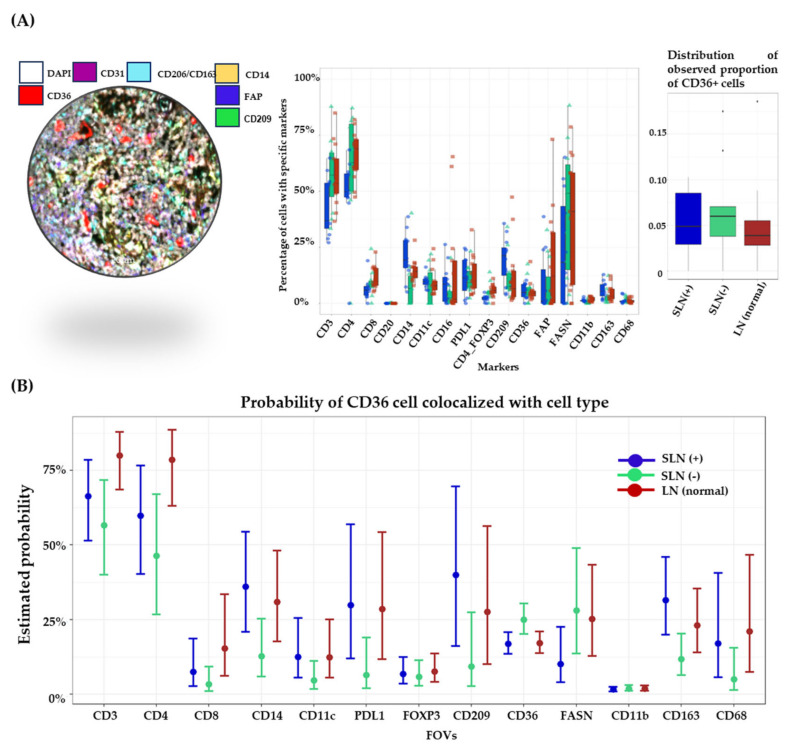
Spatial MxIF image analysis of SLN (−), SLN (+) tissue from patients, and LN tissue from control subjects. (**A**) Box plot showing the percentage of cells expressing a specific marker of all cells in a field of view (FOV) (center panel) and proportion of CD36+ cells per FOV in the three groups of samples. (**B**) Estimated probability of CD36 cells colocalized with other immune cells (within a 40-micron radius of a CD36 cell) in an analyzed panel of markers using spatial regression models.

The SKCM dataset provides prime evidence of a positive connection between CD36 and numerous immunosuppressive cells. These cues encourage us to examine the expression of CD36 and other immunosuppressive markers in histopathological slides of LN tissue from four melanoma patients and two normal LN from non-cancerous individuals. Our goal for the analysis was to detect early alterations in the LN before metastasis. Among these samples, SLN (−) is considered to have signals from primary melanoma tumors through LEVs, but SLN (+) has direct contact with LN cells and with melanoma cells. We chose multiple FOVs (*n* = 36 annotated and classified) from two samples, each from LN (normal), SLN (−), and SLN (+), and leveraged an MxIF spatial analysis to understand whether CD36 is overexpressed in the presence of tumor cells in SLN+ or EVs itself directed modification in SLN (−) tissue. We used CD3, CD4, CD8, CD14, CD11C, PDL1, FOXP3, CD209, CD36, FASN, CD11B, CD163, and CD68 markers to analyze the co-expression and colocalization of CD36 with these immune markers (Table S2). Our prior data from the in vitro experiment indicate that EVs could be the most critical players in boosting CD36 expression in monocytic and other key LN resident cells. Our data from the spatial analysis showed that immunosuppressive markers like CD163, CD209, and PDL1 were upregulated in the SLN (+) and SLN (−) of melanoma patients, which were mostly expressed on myeloid cells (Figure 4A). However, T cells, especially CD8-T cells, are downregulated in melanoma’s SLN compared to normal LN, which provides direct evidence of the immunosuppressive characteristics of SLN (+) or SLN (−) compared to normal LN (Figure 4A). CD36 expression was analyzed as a higher observed proportion in the melanoma SLNs than normal LN (Figure 4A). In the spatial regression model comparing the CD36 expression across groups, cells from melanoma SLN (+) were found to have higher odds of expressing CD36 compared to cells from SLN (−) and normal LN (OR = 1.45 and 1.06, respectively), but these comparisons were not statistically significant (Table S3). To assess whether these upregulated molecules have a direct colocalization of CD36, we used the spatial regression models to obtain estimates of the probability of CD36 cells being colocalized with each immune marker. Our results indicate that several immunosuppressive cell markers like CD209 and CD163 were more colocalized with CD36 in SLN (−) compared to control. SLN (−) are uninvolved in tumor LN, which is more likely to receive melanoma-derived EVs, and these data support that EVs could target myeloid cells (Table 1). We also found a difference in the colocalization of CD36 with CD209 and CD163 in SLN (−) and SLN (+) (Supplementary Table S4). However, these differences were not statistically significant after an adjustment for multiple comparisons (Table S4). 

## 4. Discussion

The overexpression of CD36 on tumor and immune cells implicates tumor progression and manifests poor clinical outcomes in many cancers. Our study indicates that the overexpression of CD36 is associated with decreased progression-free survival in melanoma, and the cancer hallmark enrichment dataset shows that CD36 is associated with reprogramming energy metabolism and tissue invasion or metastasis (Figure 1). CD36 serves as an important target for melanoma as it overexpresses in drug-tolerant cells of BRAF or MEK therapies [37]. More importantly, CD36 is a significant regulator of tumor growth, metastasis, and angiogenesis [5,7,38]. Data acquired in preclinical models of various malignancies indicate that inhibiting CD36 may effectively halt metastatic spread and serve as a predictive biomarker in cancer [22]. CD36, a protein involved in fatty acid metabolism, is upregulated in cancer and associated with LN metastases [21]. Deregulated fatty acid metabolism is an essential driver of metastasis [13]. Moreover, the mechanism of upregulation of CD36 in the LN has not been delineated. 

CD36 is known as a bad prognostic marker in cancer [22], and it is also associated with adverse clinicopathological features in many cancers [39]. Hence, CD36 is an emerging target for cancer therapy. Melanoma secretory factors are critical to modulating the lymphatic remodeling of the tumor-draining LN. Our laboratory has previously reported that melanoma EVs carried several proteomic cargoes involved in immunosuppression in the SLN of melanoma patients [19]. Our present study has provided evidence that melanoma-derived EVs can upregulate the CD36 expression in the recipient cells. Further, we also found that the lymphatic fluid of SLN-afferent tubules from melanoma patients carries more CD36 cargoes in LEVs than in control subjects. A melanoma-cell-line-based EV analysis also indicated the presence of CD36 cargoes in the form of mRNA and proteins. Melanoma-associated EVs are associated with the SLN premetastatic niche. In the spatial analysis of SLN (+) tissue, we detected the highest level of CD36 compared to SLN (−), and SLN (−) was higher than normal LN (Figure 4A). This shows that tumor-secreted factors play a role in upregulating CD36 in SLN prior to metastasis. In vitro data showed that melanoma-derived EVs could substantially upregulate CD36 expression in monocytic and endothelial cells. A melanoma patient’s LEVs carry significantly higher CD36 cargoes compared to a control human LEVs. This suggests that melanoma cells synthesize more CD36 to prepare the SLN “soil” for metastasis. 

CD36 within the TME has a distinct role in the vascular endothelium, tumor-associated macrophages, myeloid-derived suppressor cells (MDSCs), regulatory T (Treg) cells, and CD8+ T cells in tumor growth. We utilized bioinformatic approaches to correlate the expression in SKCM datasets and also leveraged the MxIF spatial platform to correlate immunosuppressive markers with CD36. SKCM data provide direct evidence of the strong correlation between CD163 and CD209, and the infiltration of M2 macrophages in the metastatic melanoma tumor (Figure 1). 

The macrophage polarization assay provides evidence that the CD36 cargo in the melanoma EVs determines the fate of immunosuppression in the macrophages. CD36 is an important receptor that allows entry to not only lipid particles but also facilitates the entry of lipid-enriched vesicles from the tumor, prompting these cells to initiate their tumor-promoting activities [24]. Tumor-derived-EV-exposed monocytes accumulate lipids as lipid droplets through upregulating CD36, as reported earlier [24,40]. This implies that CD36 is a critical molecule in regulating macrophage function. To verify this, we challenged melanoma-derived EVs to HLEC- and PMA-stimulated THP1 cells, presumably M0 macrophage, and these cells upregulate CD36 after challenging. Our results show that SKMEL28-derived-EV-challenged M0 macrophages can mediate functional changes, which suggests that melanoma EVs could be a modulator of macrophage functioning. Furthermore, challenging EV-derived CD36-intact melanoma cells and CD36-silenced immune cells revealed a difference in total CD36 levels in EV-challenged THP1 cells. This suggests melanoma-derived EVs’ mRNA and cargo proteins can regulate CD36 expression in target cells in response to EV challenge. There is also a possibility that melanoma-derived EVs may contain some other regulatory component that can modulate the expression of CD36 in targeted cells. Our work further supports the notion that LN macrophages are central to the generation of a premetastatic niche in the SLN, which could be facilitated by melanoma EV-based activation and CD36 overexpression. Moreover, engineered EVs can also be utilized as a potent tool for cancer treatment [41]. EVs have been used to deliver antigens to dendritic cells and prim T-cell responses against melanoma. Specifically, EVs induced with cytochalasin B and loaded with GM-CSF may provide a novel approach for presenting tumor antigens to dendritic cells [42]. 

Melanoma is an aggressive form of cancer that necessitates the development of a new cell-based immunotherapy due to the failure of successful treatment with conventional chemotherapy and radiotherapy. Multiple clinical trials have demonstrated promising results by utilizing ex vivo immune maturation and then reintroducing the matured immune cells back into patients. Immunogenic drug treatments in melanoma patients have also shown promising results. Most of these therapies focus on T-cell-, B-cell-, NK-cell-, dendritic-cell-, and macrophage-based therapy [43]. To understand EVs’ role in SLN immunosuppression, we employed numerous cellular markers to understand the impact of EVs on LN residents’ cells of control and melanoma patients that govern the development of the protumorigenic microenvironment (Table 1). Our data indicate that SLN (+) FOVs have a higher level of CD163, CD209, CD11c, CD14, PDL1, and CD36 compared to SLN (−) and control LN; however, the CD8, CD4, and CD3 expression was downregulated in SLN (+) which is primarily important for regulating antitumor LN immunity (Figure 4A). CD36 is considered a surrogate parameter for LN metastasis and risk stratification [21]. To analyze the tissue expression of CD36 and its association with other LN resident cells, we analyzed the tumor-involved LN (SLN+) and tumor-uninvolved LN (SLN−) and compared them to the normal one using a spatial MxIF analysis. The spatial MxIF analysis of LN demonstrated that an altered CD36 expression could prepare a tolerogenic microenvironment in the SLN for metastatic colonization. The colocalization of CD36 with immunosuppressive markers in melanoma cancer cells differed in SLN (−) and SLN (+), which differentiate the direct role of the tumor in generating a CD36-mediated effect in the neighborhood cells (Figure 4B). Our colocalization modeling of MxIF data to investigate the connection of CD36 with immunological markers provided insight into the deregulation of CD36 with immune cells in subsets of different SLNs of melanoma. We estimated a higher colocalization of CD36 with CD209 and CD163 in SLN (+) compared to SLN (−); however, across all spatial modeling of MxIF data, we did not find statistically significant results due to the small sample size and large number of tests performed for the exploratory analysis. Despite this limitation, our preliminary findings concur with the hypothesis of melanoma EVs promoting the LN’s immunosuppressive niche and provide evidence that this should be further investigated on a larger sample size.

## 5. Conclusions

**Figure 5:**
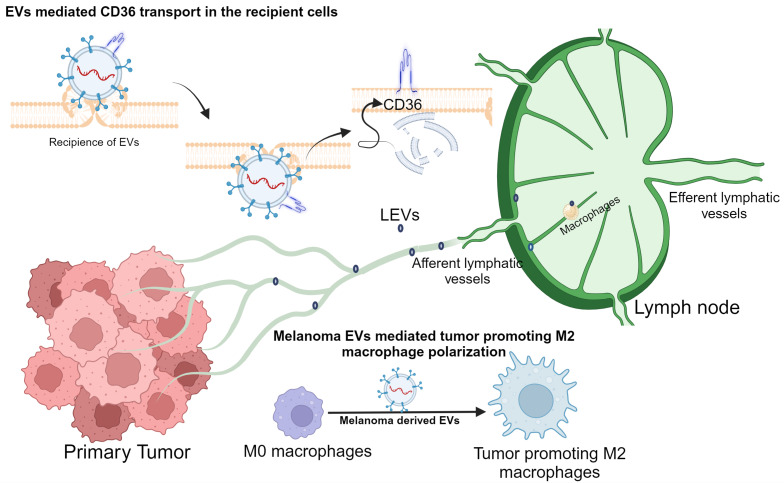
Primary-melanoma-secreted EVs in lymphatic fluid enter the SLN via afferent lymphatic vessels. The SLN components, including macrophages and endothelial cells, receive these EVs to upregulate CD36, possibly through CD36 cargoes (mRNA and protein). The upregulation of CD36 in the targeted cells can mediate the immunosuppression mechanism to promote the tumor-promoting niche in the SLN.

Previous studies have shown that CD36 promotes tumor formation, metastasis, and treatment resistance through various molecular mechanisms [39]. Our study highlights the significance of CD36 in promoting the development of a premetastatic niche in tumor-draining LN or SLN (Figure 5). Melanoma-cell-secreted EVs are capable of enriching CD36 in recipient myeloid cells, leading to immunosuppression. From the in vitro assays and spatial imaging analyses, we found evidence that CD36 polarizes immunosuppressive macrophages and the colocalization of other immune cells. Given the significance of CD36 in its involvement in premetastatic niche development and cancer cell metastasis, a new therapy line against CD36 could directly impact melanoma care. However, more preclinical testing in different biological models with various classes of CD36 inhibitors is warranted. 

## Figures and Tables

**Table 1:** CD36 cell colocalization spatial regression model results of melanoma SLN (−) compared to control LN.

Comparison	Cell Type	Odds Ratio	95% CI for OR	*p*-Value	FDR-Adjusted *p*-Value
Melanoma SLN (−) vs. Control LN	CD3	0.33	(0.13, 0.8)	0.0148	0.1153
	CD4	0.24	(0.07, 0.74)	0.0134	0.1153
	CD8	0.19	(0.04, 0.84)	0.0292	0.1576
	CD14	0.33	(0.11, 0.99)	0.0489	0.1732
	CD11C	0.35	(0.1, 1.25)	0.1046	0.2239
	PDL1	0.17	(0.03, 0.89)	0.0364	0.1576
	FOXP3	0.74	(0.28, 1.98)	0.5493	0.7651
	CD209	0.27	(0.05, 1.6)	0.149	0.2767
	CD36	1.62	(1.11, 2.34)	0.0114	0.1153
	FASN	1.16	(0.34, 3.91)	0.8141	0.9071
	CD11B	0.98	(0.5, 1.91)	0.9529	0.978
	CD163	0.44	(0.18, 1.08)	0.0728	0.2027
	CD68	0.2	(0.03, 1.11)	0.0655	0.1966

## Data Availability

The data generated or analyzed in the study are included in the published articles and supplement information. The data are available upon request from the corresponding author.

## Supplementary Materials

The following supporting information can be downloaded at: https://www.mdpi.com/article/10.3390/biom14070837/s1, Supplementary Figure S1: Differential expression of CD36 in multiple cancers; Figure S2: Timer 2.0-based analysis of CD36 with tumor, endothelial cells, and macrophages in SKCM dataset; Figure S3: NTA analysis of control and melanoma patient lymphatic EVs; Figure S4: Scatterplot with a nanoscale flow cytometer for analysis of patient and control LEVs; Figure S5: Challenging SKMEL28 EVs increases CD36 expression in THP1 cells after 48 h of incubation; Figure S6: SKMEL28 EVs upregulate CD36 expression in HLEC cells after 16 h of incubation; Figure S7: Gating strategy for CD36 expression analysis; Figure S8: Western blot analysis of NTC- and CD36-siRNA-transfected SKMEL28 cells; Figure S9: EVs collected from SKMEL28 cells transfected with NTC and siRNAs; Figure S10: Challenging C32TG EVs from NTC and SiRNA#3 shows modulation of M2-macrophage-like characteristics in PMA-pretreated THP1 cells; Supplementary Table S1: Antibodies for the MxIF panel and Table S2: Cell classification model for statistical analysis with selected markers; Supplementary Table S3: CD36 cell expression spatial regression model results; and Supplementary Table S4: CD36 cell colocalization spatial regression model results in SLN (+) compared to SLN (−) and Control LN. Original images of Figure 2A,C can be found in Supplementary Materials.
