# Supplementary material for: Melanoma-Derived Extracellular Vesicles Induce CD36-Mediated Pre-Metastatic Niche
Source: Biomolecules. 2024 Jul 11;14(7):837. doi: 10.3390/biom14070837 (PMC11275097; doi:10.3390/biom14070837)
Supplement: Supplementary file 1 [file biomolecules-14-00837-s001.zip › Supplementary Figures.pptx]

## Slide 1
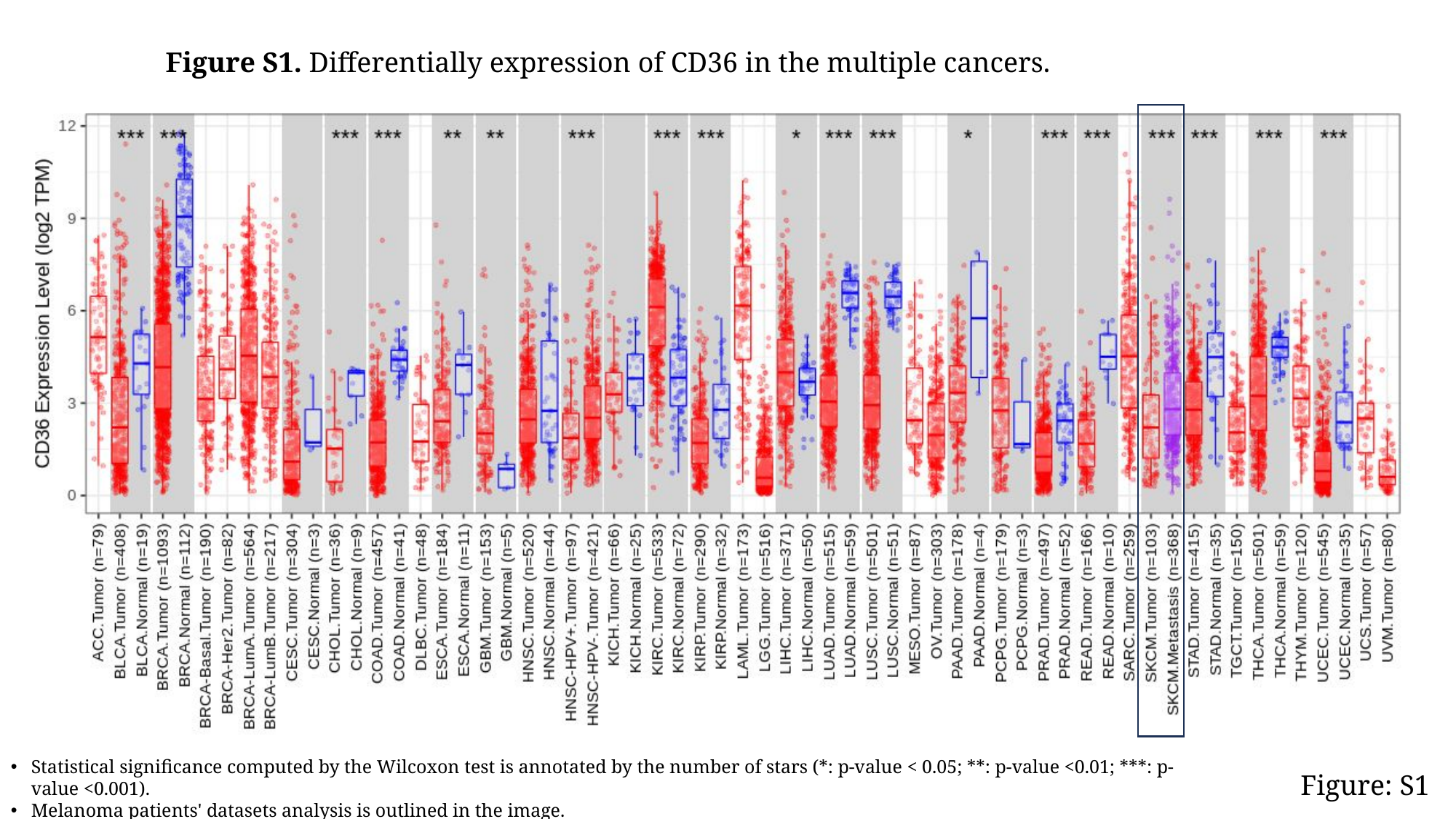

Figure S1. Differentially expression of CD36 in the multiple cancers.
Statistical significance computed by the Wilcoxon test is annotated by the number of stars (*: p-value < 0.05; **: p-value <0.01; ***: p-value <0.001).
Melanoma patients' datasets analysis is outlined in the image.
Figure: S1

## Slide 2
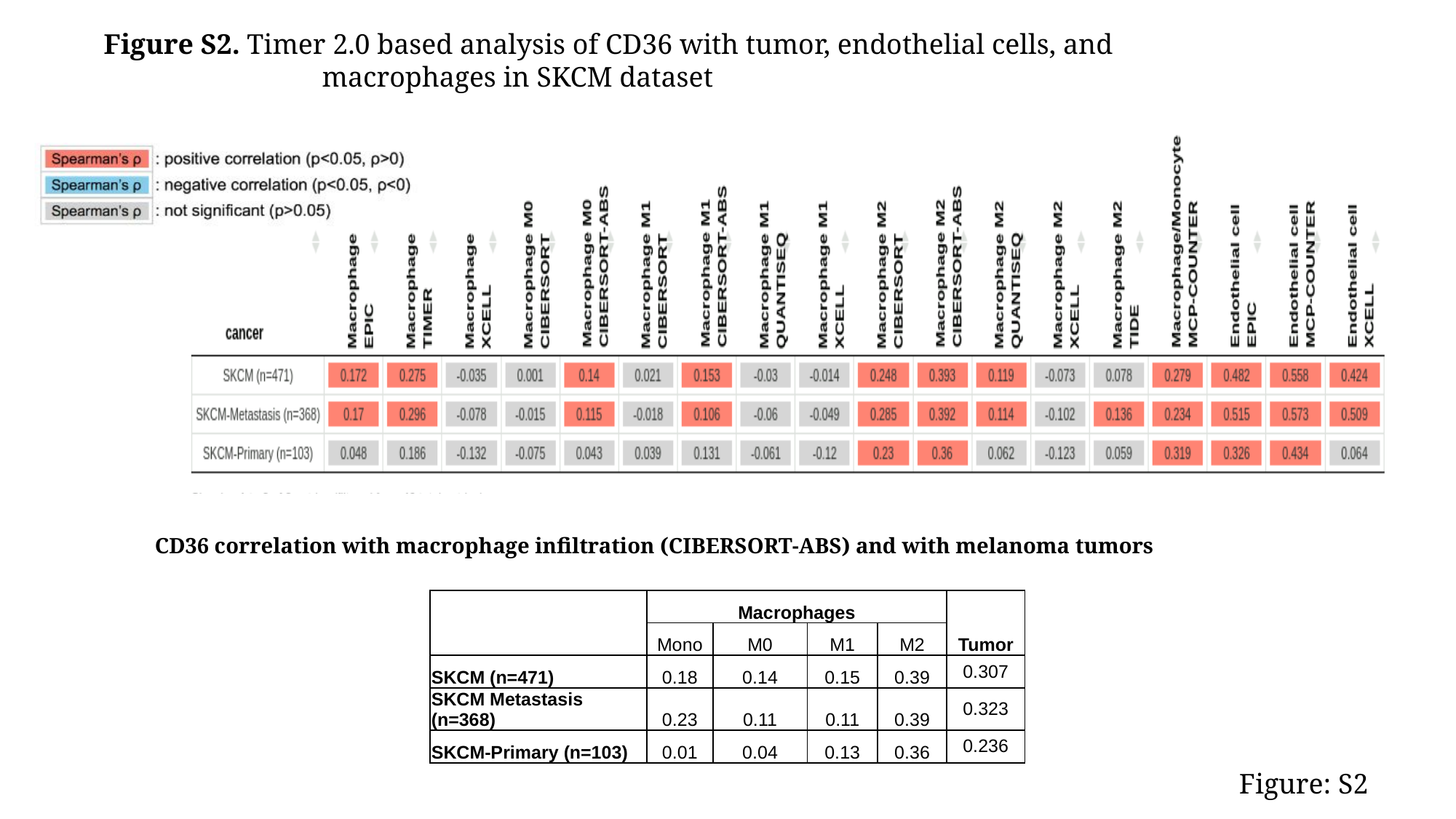

Figure S2. Timer 2.0 based analysis of CD36 with tumor, endothelial cells, and 				macrophages in SKCM dataset
CD36 correlation with macrophage infiltration (CIBERSORT-ABS) and with melanoma tumors
| | Macrophages | | | | Tumor |
| --- | --- | --- | --- | --- | --- |
| | Mono | M0 | M1 | M2 | Tumor |
| SKCM (n=471) | 0.18 | 0.14 | 0.15 | 0.39 | 0.307 |
| SKCM Metastasis (n=368) | 0.23 | 0.11 | 0.11 | 0.39 | 0.323 |
| SKCM-Primary (n=103) | 0.01 | 0.04 | 0.13 | 0.36 | 0.236 |
Figure: S2

## Slide 3
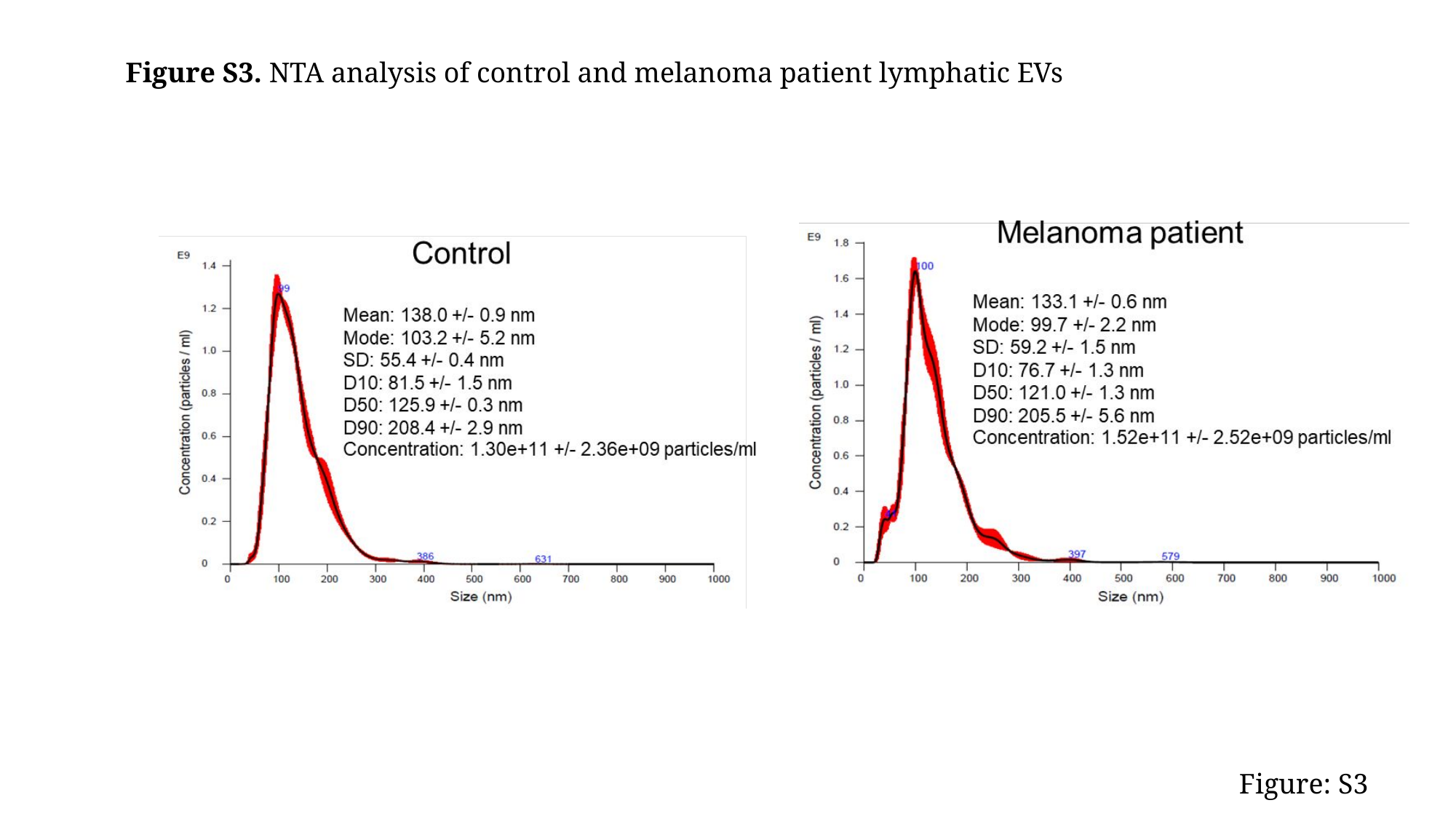

Figure S3. NTA analysis of control and melanoma patient lymphatic EVs
Figure: S3

## Slide 4
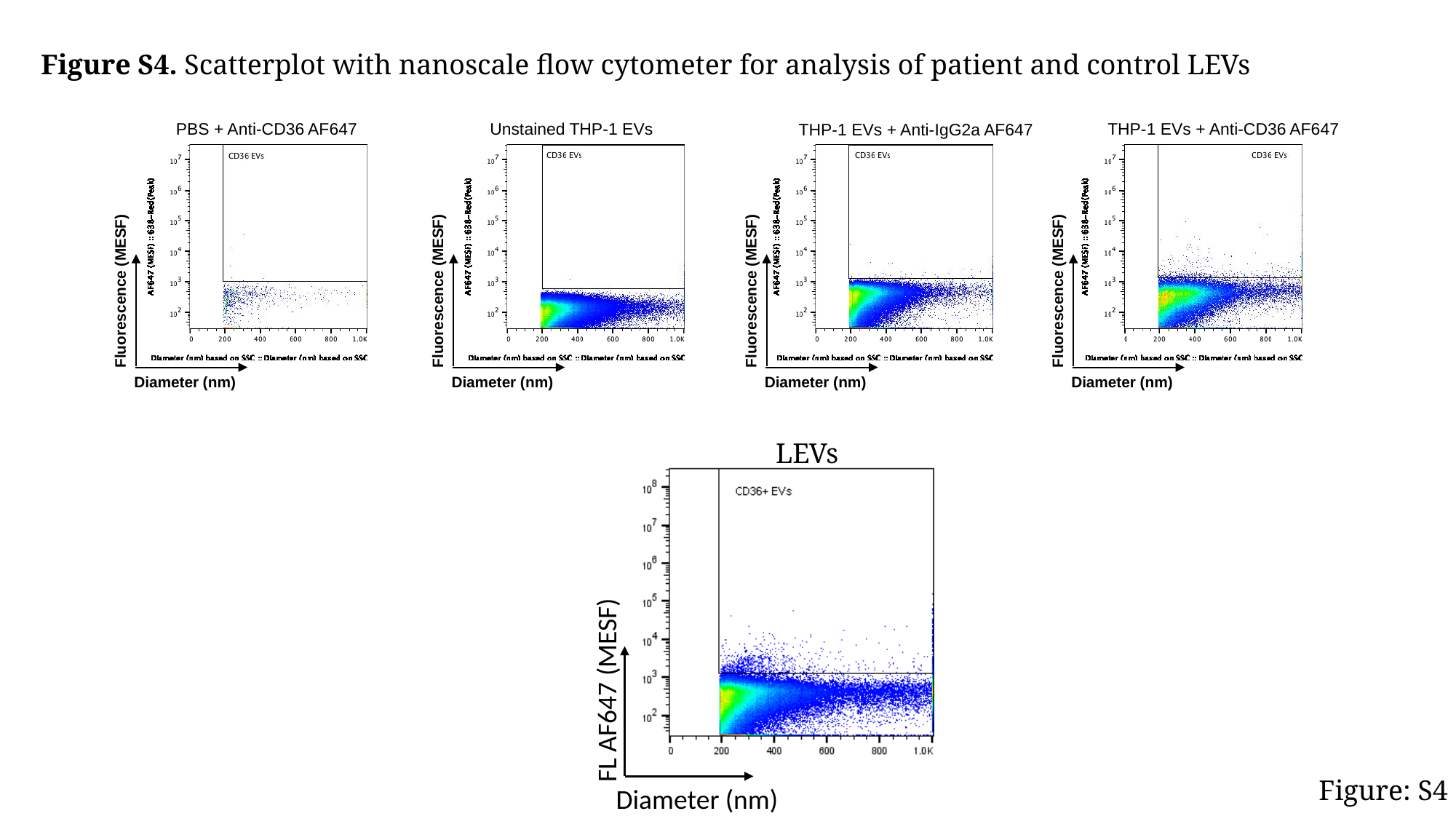

Figure S4. Scatterplot with nanoscale flow cytometer for analysis of patient and control LEVs
THP-1 EVs + Anti-CD36 AF647
PBS + Anti-CD36 AF647
Unstained THP-1 EVs
THP-1 EVs + Anti-IgG2a AF647
Fluorescence (MESF)
Diameter (nm)
Fluorescence (MESF)
Diameter (nm)
Fluorescence (MESF)
Diameter (nm)
Fluorescence (MESF)
Diameter (nm)
LEVs
FL AF647 (MESF)
Diameter (nm)
Figure: S4

## Slide 5
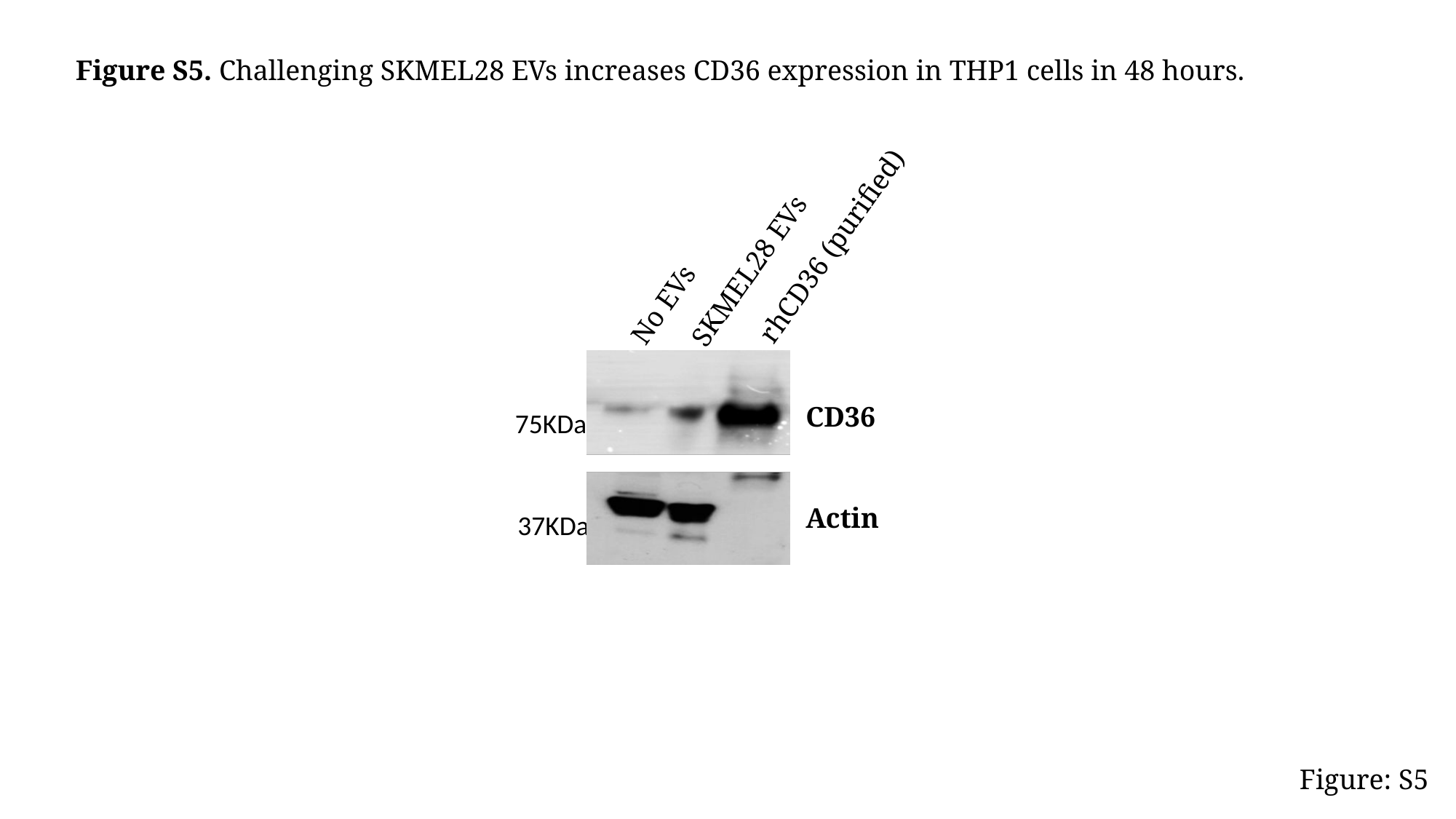

Figure S5. Challenging SKMEL28 EVs increases CD36 expression in THP1 cells in 48 hours.
rhCD36 (purified)
No EVs
SKMEL28 EVs
CD36
75KDa
Actin
37KDa
Figure: S5

## Slide 6
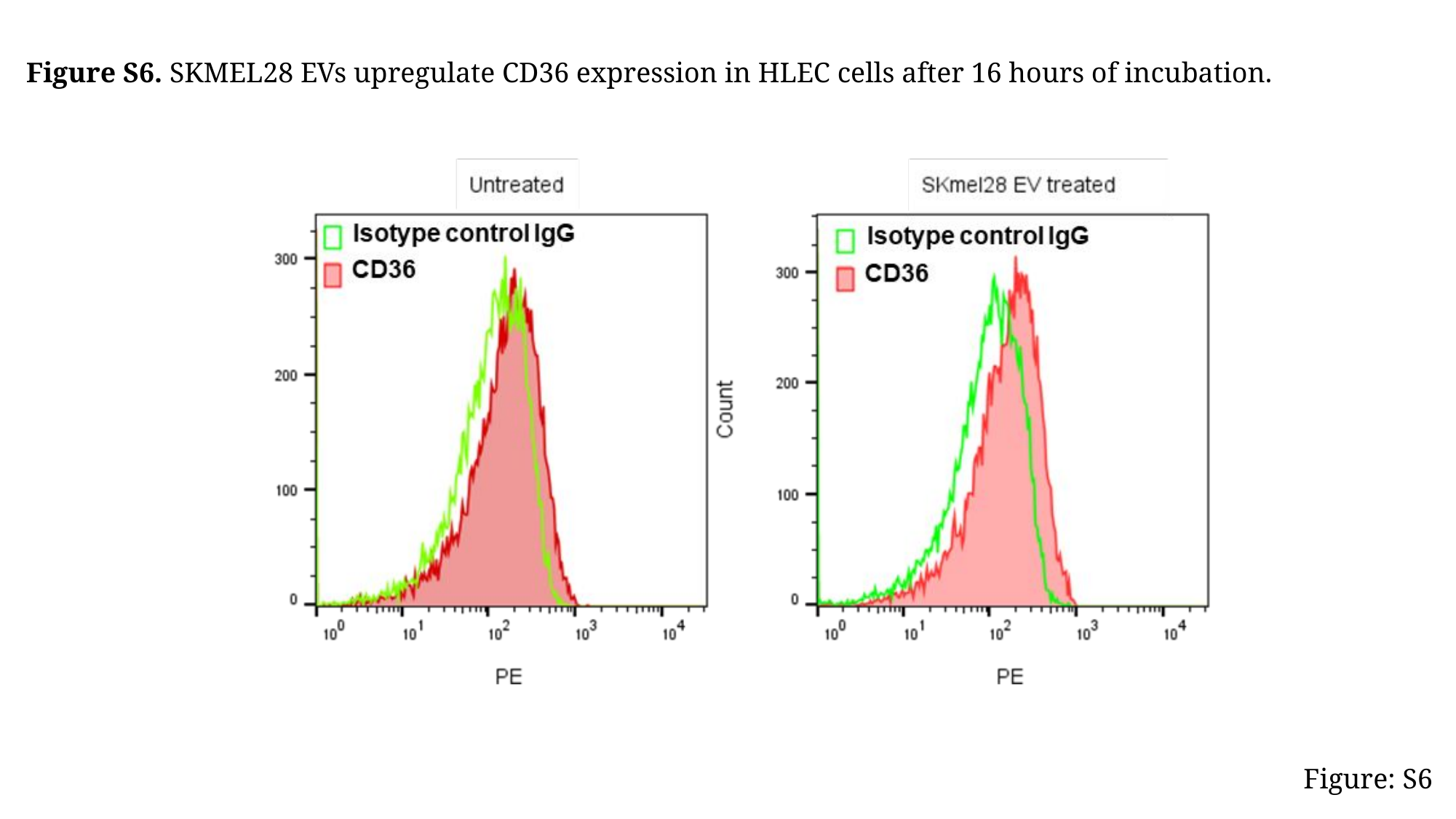

Figure S6. SKMEL28 EVs upregulate CD36 expression in HLEC cells after 16 hours of incubation.
Figure: S6

## Slide 7
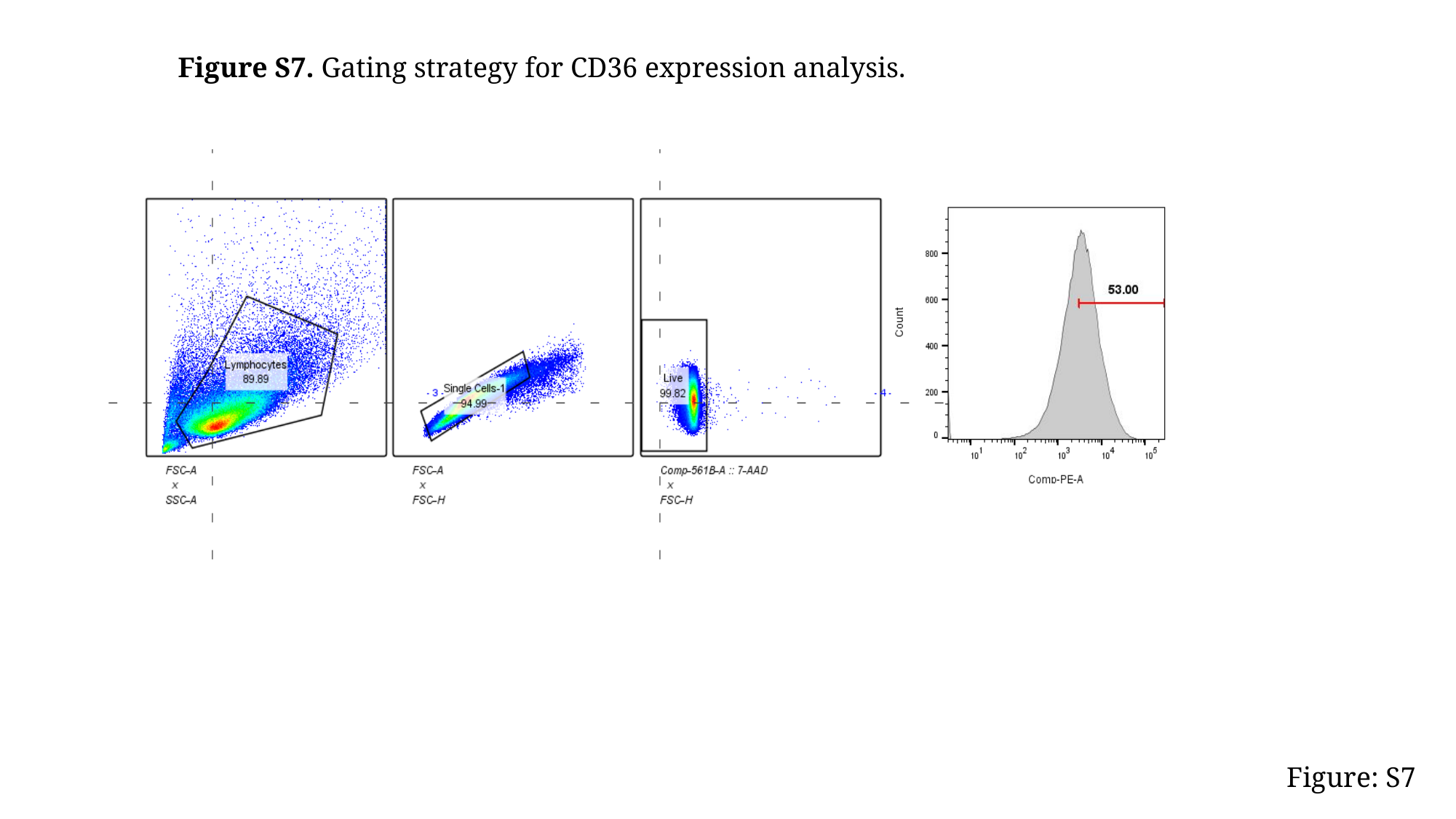

Figure S7. Gating strategy for CD36 expression analysis.
Figure: S7

## Slide 8
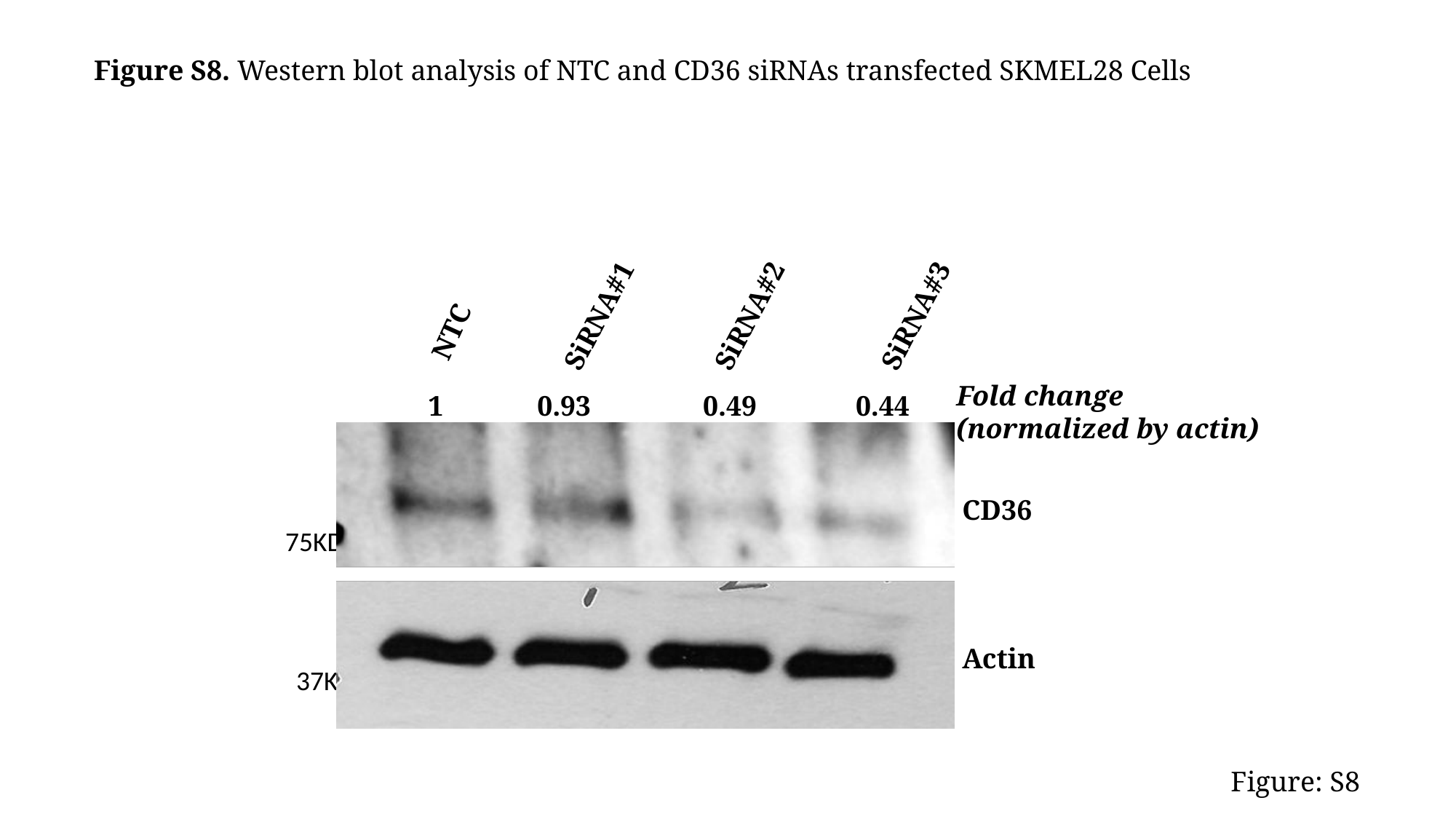

Figure S8. Western blot analysis of NTC and CD36 siRNAs transfected SKMEL28 Cells
SiRNA#1
SiRNA#2
SiRNA#3
NTC
Fold change
(normalized by actin)
1 	0.93	 0.49 0.44
CD36
75KDa
Actin
37KDa
Figure: S8

## Slide 9
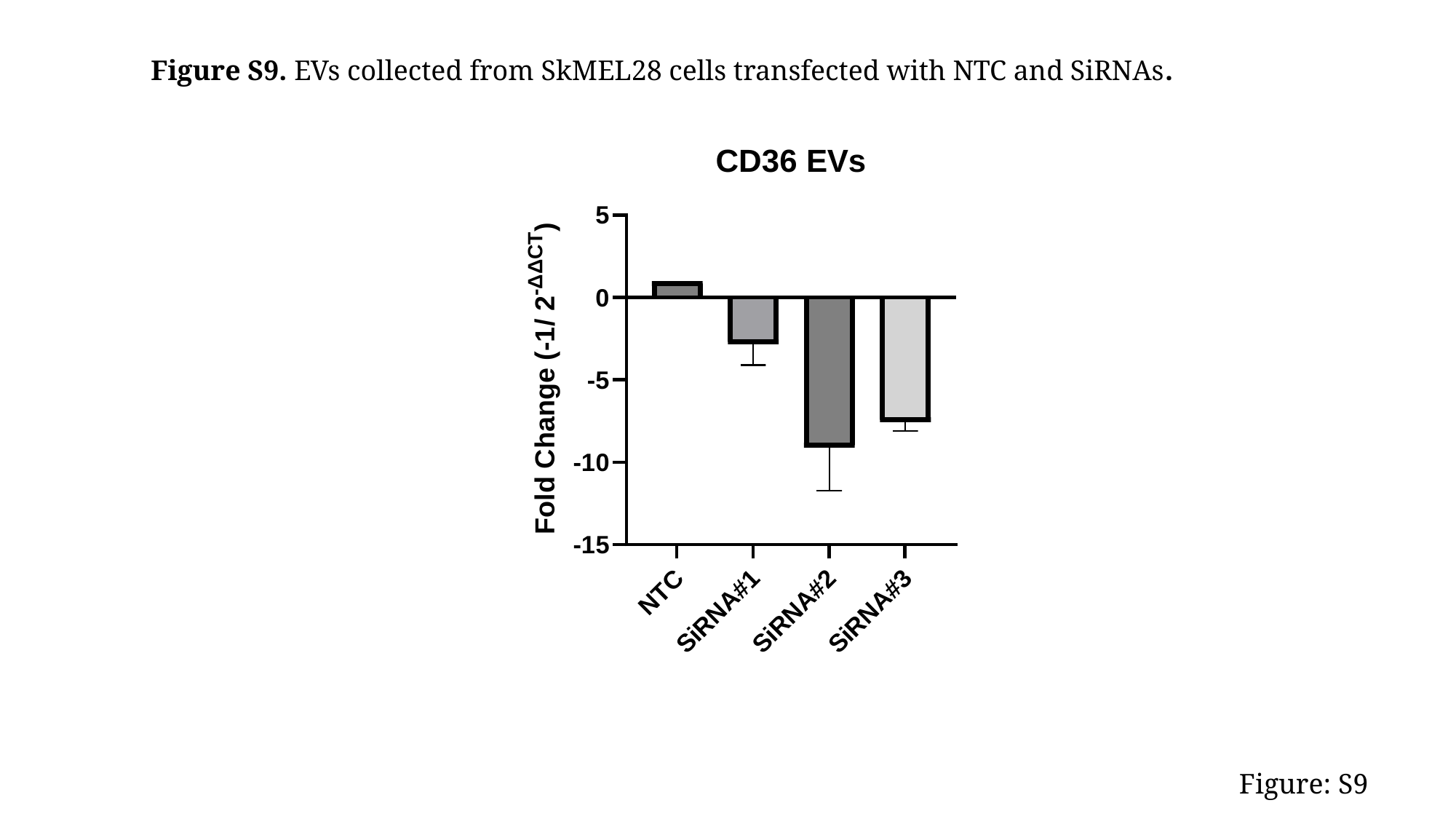

Figure S9. EVs collected from SkMEL28 cells transfected with NTC and SiRNAs.
Figure: S9

## Slide 10
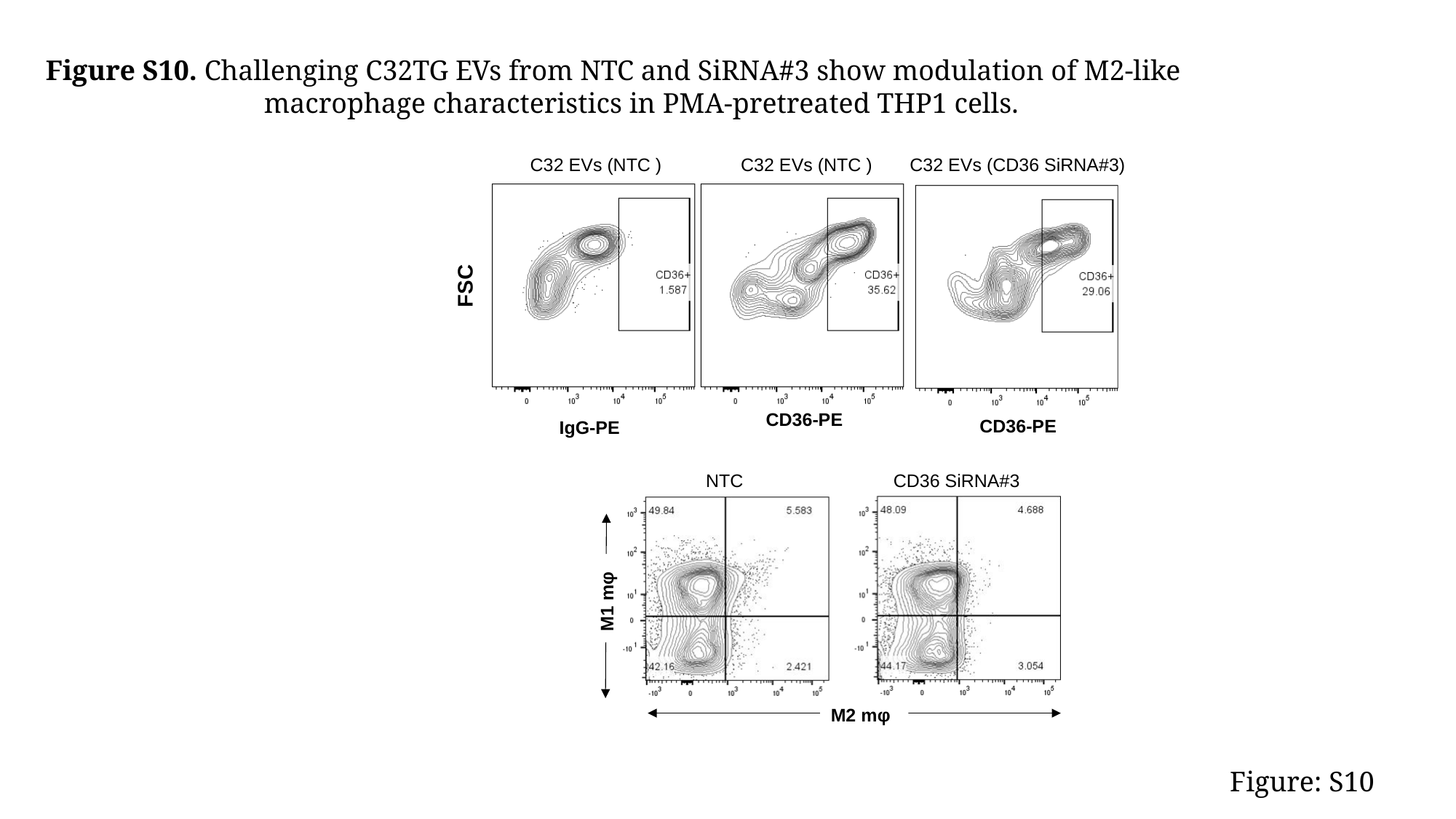

Figure S10. Challenging C32TG EVs from NTC and SiRNA#3 show modulation of M2-like 				macrophage characteristics in PMA-pretreated THP1 cells.
C32 EVs (NTC )
C32 EVs (NTC )
C32 EVs (CD36 SiRNA#3)
FSC
CD36-PE
CD36-PE
IgG-PE
CD36 SiRNA#3
NTC
M1 mφ
M2 mφ
Figure: S10

## Slide 11
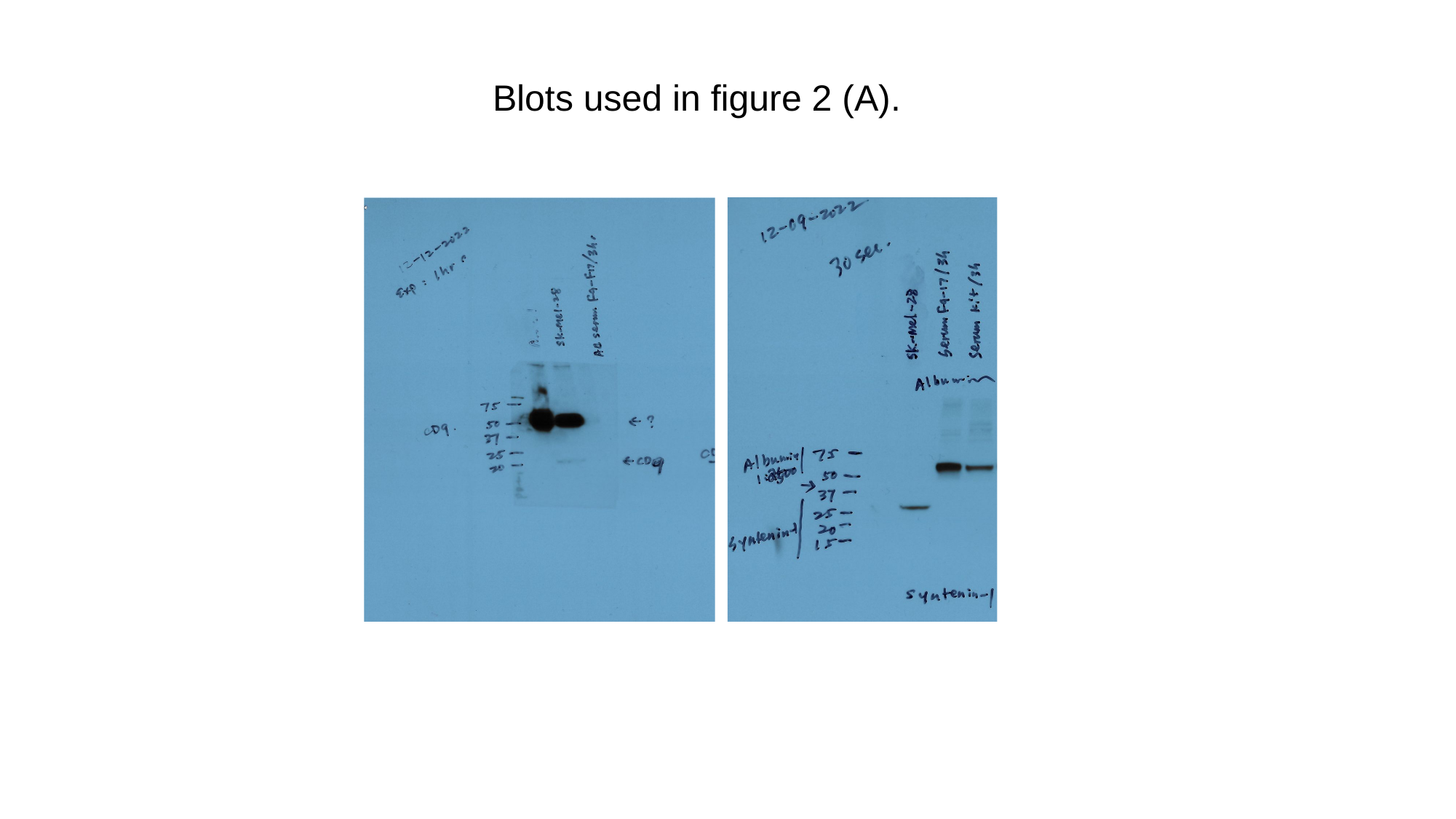

# Blots used in figure 2 (A).

## Slide 12
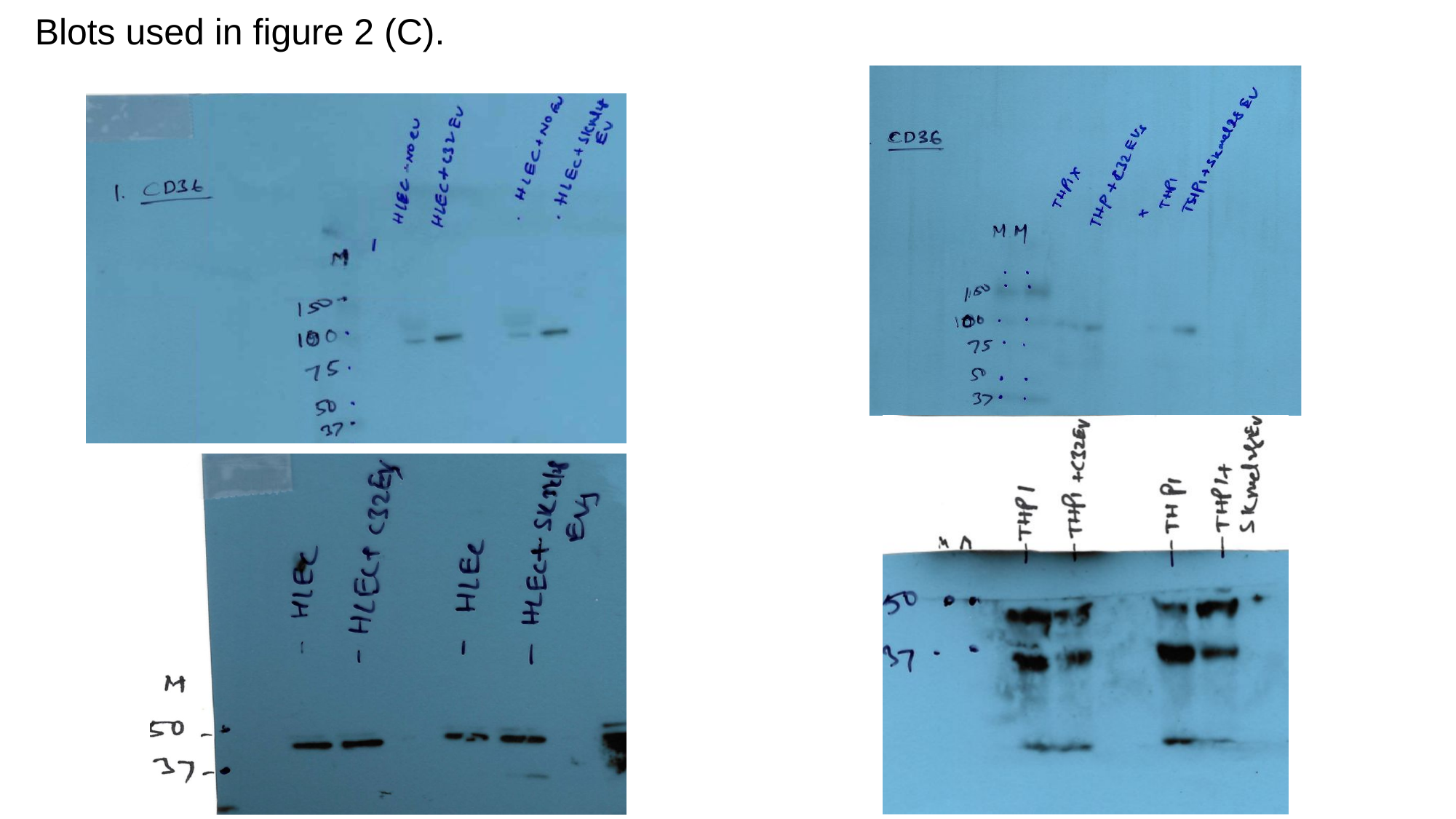

# Blots used in figure 2 (C).
