# Supplementary material for: Melanoma-Derived Extracellular Vesicles Induce CD36-Mediated Pre-Metastatic Niche
Source: Biomolecules. 2024 Jul 11;14(7):837. doi: 10.3390/biom14070837 (PMC11275097; doi:10.3390/biom14070837)
Supplement: Supplementary file 1 [file biomolecules-14-00837-s001.zip › Supplementary Tables.pdf]

**Table S1**. Antibodies for the MxIF panel.

| Conjugate | Antibody name | Company | Catalog |
|---|---|---|---|
| Cy3 | CD163 | Bio-rad | MCA1853 |
| Cy3 | CD4 | abcam | ab181724 |
| Cy3 | CD11C | abcam | ab216655 |
| Cy3 | MPO | abcam | ab221847 |
| Cy5 | NaKATPase | abcam | ab76020 |
| Cy5 | FOXP3 | abcam | ab96048 |
| Cy5 | PDL1 | abcam | ab221612 |
| Cy5 | CD3 | abcam | ab17143 |
| Cy5 | CD16 | abcam | ab215977 |
| Cy3 | CD11b | abcam | ab238794 |
| Cy5 | CD14 | abcam | ab214438 |
| Cy5 | FASN | Cell Signaling | 66058 |
| Cy5 | CD68 | Epredia | MS-397P |
| Cy5 | CD36 | Cell Signaling | CS39914SF |
| Cy3 | CD38 | abcam | ab204940 |
| Cy5 | FAP | R&D Systems | AF3715 |
| Cy3 | CD209 | abcam | ab218883 |
| Cy3 | CD8 | Novus | NOVUA2PABX210601 |

**Table S2**. Cell classification model for statistical analysis with selected markers.

| Surface markers | Cell types/Functional markers |
|---|---|
| CD3, CD4, CD8 | T Cells |
| CD14, CD16, CD11c, CD209, CD206, CD163, CD68, | Macrophages |
| CD36, FASN, PDL1, FOXp3 | Functional markers |
| CD20 | B-Cells |
| MPO | Neutrophils |

**Table S3.** CD36 cell expression spatial regression model results.

| Comparison | Odds ratio | 95% CI for OR | p-value |
|---|---|---|---|
| SLN (+) / SLN (-) | 1.45 | (0.25, 8.62) | 0.6881 |
| SLN (+) / Control LN | 1.06 | (0.19, 5.87) | 0.953 |
| SLN (-) / Control LN | 0.74 | (0.13, 4.25) | 0.7261 |

**Table S4.** CD36 cell colocalization spatial regression model results in SLN (+) compared to SLN (-) and Control LN.

| Comparison | Cell type | Odds ratio | 95% CI for OR | p-value | FDR adjusted p-value |
|---|---|---|---|---|---|
| SLN (+) / SLN (-) | CD3 | 1.51 | (0.61,3.76) | 0.3753 | 0.5629 |
| | CD4 | 1.72 | (0.54,5.53) | 0.3609 | 0.5629 |
| | CD8 | 2.35 | (0.52,10.63) | 0.2667 | 0.4678 |
| | CD14 | 3.84 | (1.24,11.86) | 0.0194 | 0.1259 |
| | CD11C | 2.91 | (0.8,10.6) | 0.1053 | 0.2239 |
| | PDL1 | 6.12 | (1.16,32.4) | 0.0332 | 0.1576 |
| | FOXP3 | 1.19 | (0.44,3.21) | 0.7387 | 0.8976 |
| | CD209 | 6.48 | (1.07,39.07) | 0.0416 | 0.1621 |
| | CD36 | 0.61 | (0.42,0.88) | 0.0091 | 0.1153 |
| | FASN | 0.29 | (0.08,1.07) | 0.0631 | 0.1966 |
| | CD11B | 0.85 | (0.43,1.67) | 0.6391 | 0.8308 |
| | CD163 | 3.46 | (1.41,8.48) | 0.0066 | 0.1153 |
| | CD68 | 3.9 | (0.68,22.29) | 0.1261 | 0.2458 |
| SLN (+) / Control LN | CD3 | 0.49 | (0.21,1.17) | 0.1091 | 0.2239 |
| | CD4 | 0.41 | (0.14,1.21) | 0.1065 | 0.2239 |
| | CD8 | 0.45 | (0.1,1.91) | 0.2759 | 0.4678 |
| | CD14 | 1.26 | (0.44,3.59) | 0.6689 | 0.8416 |
| | CD11C | 1.01 | (0.3,3.45) | 0.9889 | 0.9889 |
| | PDL1 | 1.06 | (0.22,5.14) | 0.9377 | 0.978 |
| | FOXP3 | 0.88 | (0.34,2.23) | 0.784 | 0.8993 |
| | CD209 | 1.74 | (0.31,9.9) | 0.5313 | 0.7651 |
| | CD36 | 0.98 | (0.68,1.41) | 0.924 | 0.978 |
| | FASN | 0.33 | (0.09,1.18) | 0.088 | 0.2239 |
| | CD11B | 0.83 | (0.44,1.58) | 0.5784 | 0.7779 |
| | CD163 | 1.54 | (0.65,3.63) | 0.3288 | 0.5343 |
| | CD68 | 0.77 | (0.14,4.18) | 0.7595 | 0.8976 |
